# Thin film encapsulation for organic light-emitting diodes using inorganic/organic hybrid layers by atomic layer deposition

**DOI:** 10.1186/s11671-015-0857-8

**Published:** 2015-04-08

**Authors:** Hao Zhang, He Ding, Mengjie Wei, Chunya Li, Bin Wei, Jianhua Zhang

**Affiliations:** Key Laboratory of Advanced Display and System Applications, Ministry of Education, Shanghai University, Yanchang Road 149, Shanghai, 200072 China; School of Mechatronic Engineering and Automation, Shanghai University, Yanchang Road 149, Shanghai, 200072 China

**Keywords:** 68.35.bm; 68.35.Ct; 68.35.Fx; 73.61.Ph, Thin film encapsulation, Atomic layer deposition, Hybrid layer, Lifetime, Organic layer thickness

## Abstract

A hybrid nanolaminates consisting of Al_2_O_3_/ZrO_2_/alucone (aluminum alkoxides with carbon-containing backbones) grown by atomic layer deposition (ALD) were reported for an encapsulation of organic light-emitting diodes (OLEDs). The electrical Ca test in this study was designed to measure the water vapor transmission rate (WVTR) of nanolaminates. We found that moisture barrier performance was improved with the increasing of the number of dyads (Al_2_O_3_/ZrO_2_/alucone) and the WVTR reached 8.5 × 10^−5^ g/m^2^/day at 25°C, relative humidity (RH) 85%. The half lifetime of a green OLED with the initial luminance of 1,500 cd/m^2^ reached 350 h using three pairs of the Al_2_O_3_ (15 nm)/ZrO_2_ (15 nm)/alucone (80 nm) as encapsulation layers.

**Electronic supplementary material:**

The online version of this article (doi:10.1186/s11671-015-0857-8) contains supplementary material, which is available to authorized users.

## Background

Active matrix organic light-emitting diodes (AM OLED) was focused as the next-generation display since its great advantages, vivid full color ,high brightness, low power consumption, fast response time, and suitable for flexible display [1]. In terms of OLEDs technology, the encapsulation process is a core technology influencing both the lifetime and reliability of OLEDs. The devices need encapsulation materials to protect it from water and oxygen. Thin film encapsulation (TFE) is considered as one of the most potent methods to ensure for protection from moisture and oxygen penetration in electronic devices [2,3]. Metal oxide thin film has been the choices for TFE materials such as aluminum oxide (Al_2_O_3_) and zirconium oxide (ZrO_2_) and titanium oxide (TiO_2_) grown by atomic layer deposition (ALD) since their superior protection forms moisture [4-6]. Recently, several researches have focused on the multilayered nanolaminate structure which comprised of alternating layer of different materials that have individual layer of nanometer-scale thickness [7,8].

However, inorganic materials have critical weaknesses such as cracking and pinhole defects in the layer surface [9,10]. To solve these problems, alternating inorganic and organic layer pairs is suggested as an encapsulation solution for OLED devices. Generally, in the multilayer structure, the role of the organic layer is known to decouple defects in the oxide layers, thereby preventing propagation of defects through the multilayer structure. ALD and molecular layer deposition (MLD) processes can deposit smooth, conformal, and pinhole-free films. Recently, an ALD/MLD combination structure has been proposed because ALD/MLD multilayers have very good film integrity which causes them to have advanced performance for thin film encapsulation [11-13].

In this study, we report on thin encapsulation layers deposited by ALD at 85°C. For the thin encapsulation layers, various nanolaminate structures consisting of Al_2_O_3_/ZrO_2_/alucone (aluminum alkoxides with carbon-containing backbones) were tested to determine the best structure producing the long lifetime devices based on the fact that nanolaminate structures significantly enhanced the lifetime by suppressing the formation of both microscopic voids and nanocrystals that could exist in an Al_2_O_3_ or ZrO_2_ single layer.

## Methods

**Figure 1:**
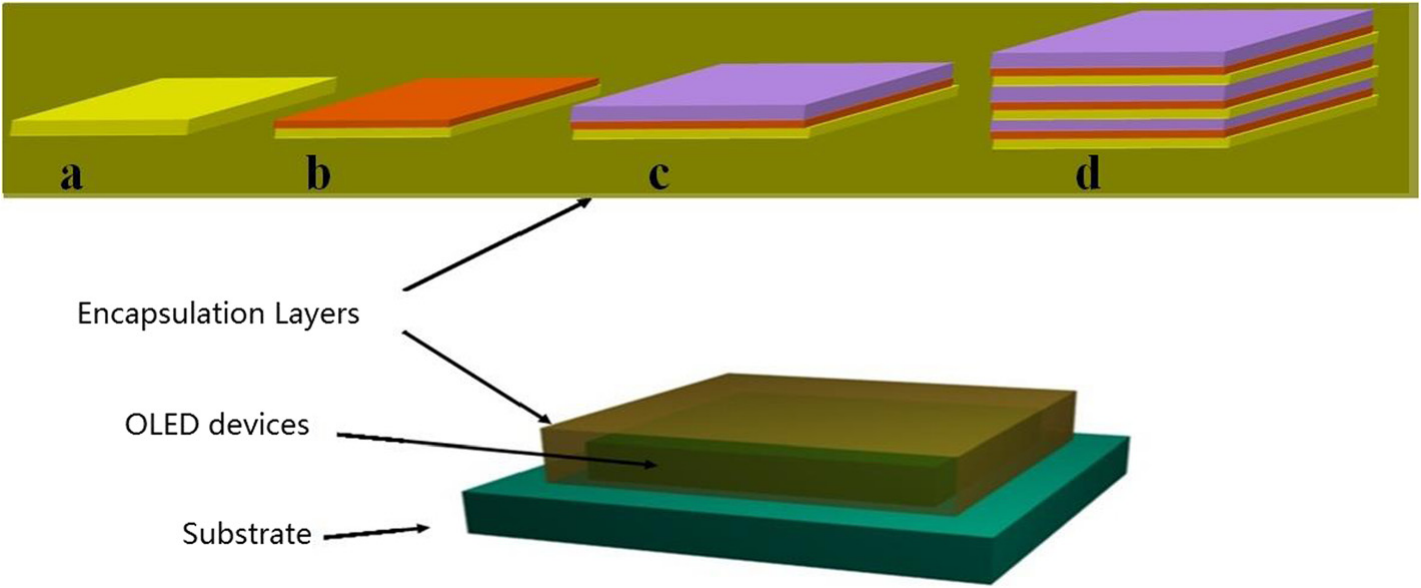
**Structure of OLED encapsulation. (a)** Single Al_2_O_3_ film devices. **(b)** Al_2_O_3_/ZrO_2_ devices. **(c)** Al_2_O_3_/ZrO_2_/alucone devices. **(d)** Three pairs Al_2_O_3_/ZrO_2_/alucone devices.

The encapsulation structure of OLEDs is shown in Figure 1, and OLED devices were fabricated by conventional vacuum deposition system. Encapsulation layers were deposited by ALD system. An indium tin oxide (ITO, 10 ohm, 150 nm)-coated glass substrates were used and cleaned with a detergent solution, deionized water, and acetone. After treated for 10 min by plasma, the substrate was transferred to vacuum deposition system. The OLEDs were fabricated by sequentially depositing the following organic layer, which were 4,4′,4″-tris-N-naphthyl-N-phenylamino-triphenylamine (40 nm) as a hole injection layer, N,N′-bis-(naphthyl)-N,N′-diphenyl-1,1′-biphenyl-4,4′-diamine (20 nm) as a hole transport layer, tris(8-hydroxyquinolinato)aluminum (Alq_3_) doped (20 nm) as a light-emitting layer, and Alq_3_(30 nm) as an electron transport layer using a shadow mask. In addition, lithium fluoride (LiF, 0.5 nm) and aluminum (Al, 100 nm) as electron injection layer and cathode, respectively, were evaporated onto the organic layer using a metal shadow mask. The typical deposition rates were 0.5 Å/s, 0.1 Å/s, and 5.0 Å/s for organic materials, LiF, and Al, respectively.

ALD Al_2_O_3_ films were fabricated using H_2_O and trimethylaluminum (TMA) as precursors at 85°C. ALD ZrO_2_ films were fabricated using H_2_O and tetrakis (dimethylamido) zirconium (TDMAZ) as precursors at 85°C. Nitrogen (N_2_, 99.999%) was used as a carrier gas on the TMA/TDMAZ and as the purge gas. Alucone films were grown using TMA and ethylene glycol (EG) at 85°C. The process pressure of ALD and MLD was 500 mTorr. In addition, Al_2_O_3_ films were deposited using 100 ms of TMA pulse, 7 s of N_2_ purge time, 100 ms of H_2_O pulse, and 7 s of purge, while ZrO_2_ films were deposited using 250 ms of TDMAZ pulse, 10 s of N_2_ purge, 200 ms H_2_O pulse, and 10 s of purge time, and alucone films were deposited using 200 ms of TMA pulse, 15 s of N_2_ purge, 300 ms of EG flow with carrier gas, and 20 s of N_2_ purge.

**Figure 2:**
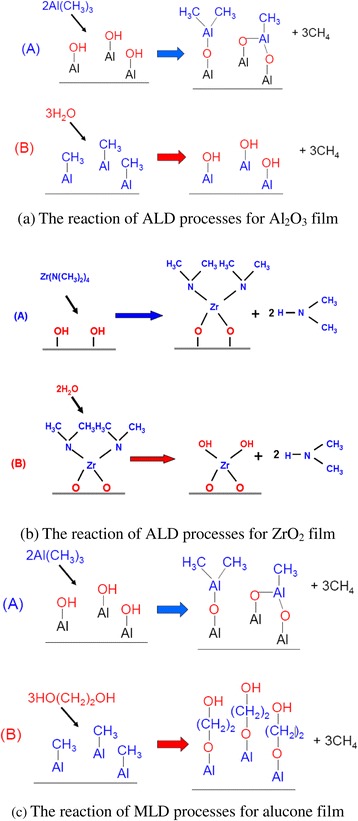
**The reaction of encapsulation film. (a)** ALD Al2O3 film reaction. **(b)** ALD ZrO2 film reaction. **(c)** MLD alucone film reaction.

The general reactions between the metal alkyl and the diol of ALD Al_2_O_3_ film and MLD alucone film can be written as follows (Figure 2). The thicknesses of the ALD film were measured by spectroscopy ellipsometry at wavelengths from 245.57 to 1,664.00 nm.

## Results and discussion

### Surface morphology

**Figure 3:**
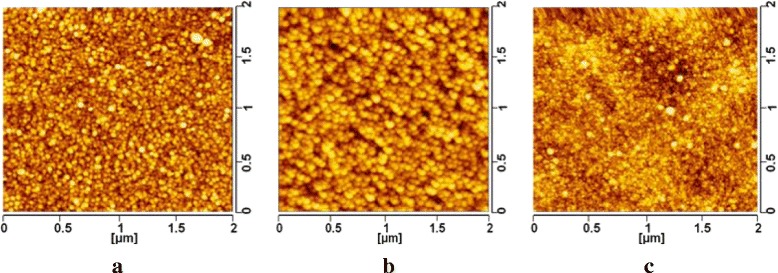
**AFM picture of thin film. (a)** Single Al_2_O_3_ film. **(b)** Al_2_O_3_/ZrO_2_ film. **(c)** Al_2_O_3_/ZrO_2_/alucone film.

We have investigated the surface morphology of the encapsulation film using atomic force microscopy (AFM) measurement with a trapping mode on the silicon wafer substrate. Figure 3a,b,c shows the surface topography of the single Al_2_O_3_, Al_2_O_3_/ZrO_2_, and Al_2_O_3_/ZrO_2_/alucone, respectively. The root-mean-square (RMS) surface roughness of the single Al_2_O_3_, Al_2_O_3_/ZrO_2_, and Al_2_O_3_/ZrO_2_/alucone layers was 1.12, 1.31, and 0.83 nm separately. The lower roughness of Al_2_O_3_/ZrO_2_/alucone film indicates that the introduction of alucone can make the surface smoother. Moreover, we have observed that the surface topography of the Al_2_O_3_ film was similar to cloth-like while that of the ZrO_2_ was grain-like, revealing that the Al_2_O_3_ layer is more suitable to deposit directly onto the cathode before the ZrO_2_ layer.

### Optical transmission

**Figure 4:**
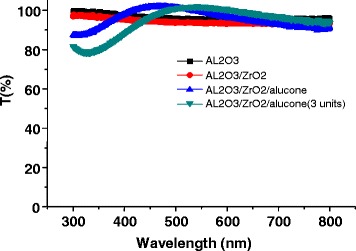
**Light transmission characteristics of the encapsulation film.**

The used Al_2_O_3_, ZrO_2_, and alucone films are highly transparent. We have measured the light transmission characteristics of the encapsulation film, as shown in Figure 4. The light transmission of the Al_2_O_3_ and ZrO_2_ layer is above 95%. In addition, it is worth to note that the light transmission of both Al_2_O_3_/ZrO_2_/alucone (one pair) and Al_2_O_3_/ZrO_2_/alucone (three pairs) are higher than those of the Al_2_O_3_ and ZrO_2_ layers at the range of 400 to 700 nm. The addition of alucone can effectively prevent the light scattering in visible wavelength (450 to 650 nm) due to the its lower surface RMS. Although the transmission below 450 nm is indeed lower than Al_2_O_3_ and Al_2_O_3_/ZrO_2_ films, the transmission increases due to the decreasing light scattering (Additional file 1: Figure S1). This demonstrates that organic/inorganic film may be suitable for the visible electroluminescent emission of the top-emitting OLEDs.

### WVTR result

**Figure 5:**
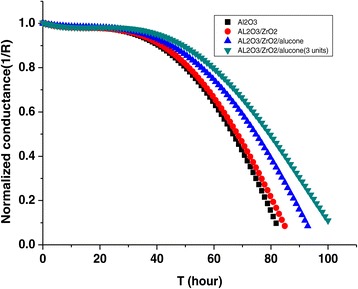
**Permeation rate measurement of ALD encapsulation.**

The Ca test in this study was designed to the water vapor transmission rate (WVTR) of nanolaminates. The amount of Ca oxidation was used to calculate the amount of water vapor using the resistivity of Ca films. We utilized the 200-nm-thick Ca layer, which is close to the normal thickness of an aluminum cathode in OLED. To prevent the Ca film from contacting with water and oxygen, the ALD system and the equipment for Ca fabrication were connected with a glove box filled with nitrogen gas. WVTR of barriers was calculated as the following equation [14-17]:

$$ p=n\frac{M_{\left(\mathrm{reagent}\right)}}{M_{\left(\mathrm{C}\mathrm{a}\right)}}\delta \rho \frac{\rho }{b}\frac{d\left(\raisebox{1ex}{$1$}\!\left/ \!\raisebox{-1ex}{$R$}\right.\right)}{dt} $$

where *n* is the molar equivalent of the degradation reaction, *M*_(reagent)_ and *M*_(Ca)_ are the molar masses of the permeating reagent and Ca, *ρ*is the Ca resistivity, and *δ* is the density of Ca. Figure 5 shows the barrier performance of different thin films. The WVTR values of ALD-grown moisture barrier films with three pairs of Al_2_O_3_/ZrO_2_/alucone are as low as 8.5 × 10^−5^ g/m^2^/day at 25°C, 85% relative humidity (RH).

### Lifetime of OLED

**Figure 6:**
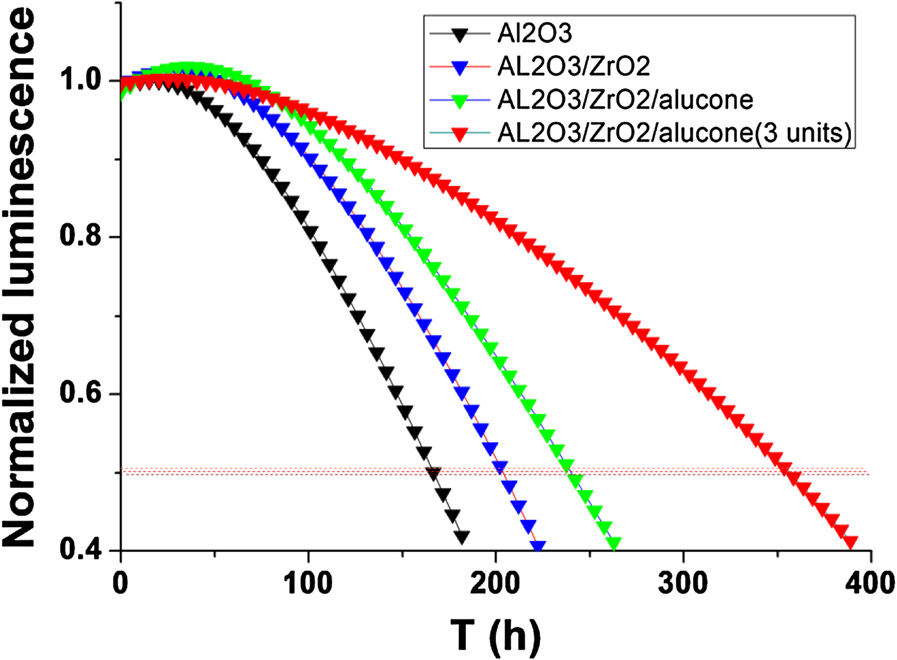
**Lifetime measurement of OLED with thin film encapsulation.**

We have measured the lifetime of OLEDs encapsulated by various ALD thin film structure. For devices A and B, the passivation film were Al_2_O_3_ (30 nm) and Al_2_O_3_ (15 nm)/ZrO_2_ (15 nm), while devices C and D used Al_2_O_3_ (15 nm)/ZrO_2_ (15 nm)/alucone (80 nm) and three pairs of Al_2_O_3_ (15 nm)/ZrO_2_ (15 nm)/alucone (80 nm). We compared the evolution of the luminance devices A, B, C, and D as shown in Figure 6. It was shown that the lifetime of device A with single Al_2_O_3_ layer decreased obviously than device B with Al_2_O_3_ (15 nm)/ZrO_2_ (15 nm). The Al_2_O_3_ layer acted as a better moisture barrier than ZrO_2_ at the same thickness. The Al_2_O_3_ layer functioned as a better moisture barrier than ZrO_2_ at the same thickness. The WVTR of Al_2_O_3_ and ZrO_2_ were 2.38 × 10^−3^ g/m^2^/day and 4.5 × 10^−3^ g/m^2^/day when fixing the film thickness by 30 nm. A denser ZrAlxOy-aluminate phase with higher packing density could be formed at the interfaces between Al_2_O_3_ and ZrO_2_, leading to a densification at the Al_2_O_3_/ZrO_2_ interfaces [18,19]. Because the permeation rate for gasses such as water vapor depends on the density of the material, even a small amount of water can enter into the device through the thin film.

Device D with three pairs of inorganic/organic hybrid layers can drastically improve the lifetime of OLED because the organic layer (alucone) may increase the water vapor diffusion path in the film and decrease the diffusion speed (or diffusivity) by trapping water vapor chemically. Generally, it is called a ‘tortuous path’, which is possibly governed by the strong dependence of the WVTR on the organic layer thickness [10,20,21]. The half lifetime of a green OLED with the initial luminance of 1,500 cd/m^2^ reached 380 h using three pairs of the Al_2_O_3_ (15 nm)/ZrO_2_ (15 nm)/alucone (80 nm) as encapsulation layers.

## Conclusions

Hybrid Al_2_O_3_/ZrO_2_/alucone thin film encapsulation structure can obviously improve barrier performance. The WVTR is as low as 8.5 × 10^−5^ g/m^2^/day at 25°C, 85% RH. A half lifetime of 380 h at initial luminance of 1,500 cd/m^2^ for a green organic light-emitting diode with developed TFE technology has been achieved.

## Additional file

Additional file 1: Figure S1. The light scattering in visible wavelength (450-650 nm) for different type of encapsulated film.

## Abbreviations

ALD: Atomic layer deposition
OLED: Organic lighting emitting diodes
WVTR: Water vapor transmission rate
RMS: Root-mean-square
