# Supplementary material for: Thin film encapsulation for organic light-emitting diodes using inorganic/organic hybrid layers by atomic layer deposition
Source: Nanoscale Res Lett. 2015 Apr 8;10:169. doi: 10.1186/s11671-015-0857-8 (PMC4402680; doi:10.1186/s11671-015-0857-8)
Supplement: Additional file 1: Figure S1. — The light scattering in visible wavelength (450-650 nm) for different type of encapsulated film. [file 11671_2015_857_MOESM1_ESM.doc]

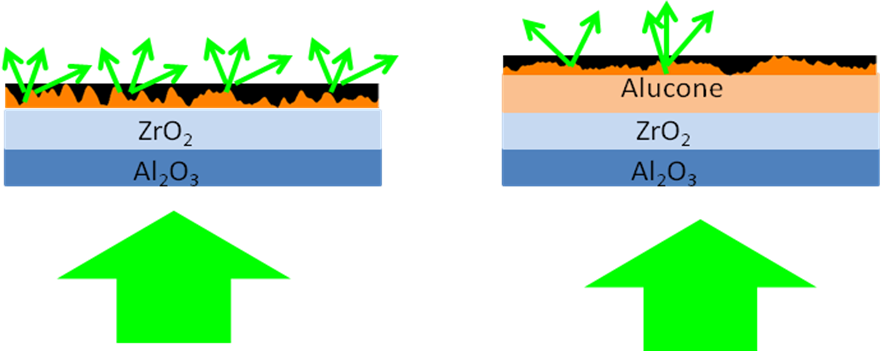


Fig. Supp. The light scattering in visible wavelength (450-650 nm) for different type of encapsulated film
